# Anesthetic Management of a 24-Year-Old Male With Tracheal Stenosis Planned for Tracheal Resection and Anastomosis: A Case Report

**DOI:** 10.7759/cureus.63424

**Published:** 2024-06-28

**Authors:** Shubhangi Humane, Sheetal Jaykar, Ipshita Garg, Shahbaz Hasnain

**Affiliations:** 1 Anaesthesiology, Dr. D. Y. Patil Medical College, Hospital and Research Centre, Dr. D. Y. Patil Vidyapeeth, Pune (Deemed to be University), Pune, IND

**Keywords:** montgomery t-tube, shared airway, guardian suture, fogarty catheter, tracheal resection and anastomosis

## Abstract

Tracheal resection and anastomosis are among the most challenging surgeries. Advancements in this field have made a variety of surgical, anesthetic, and airway management options possible. This procedure calls for multidisciplinary preoperative planning and close communication during surgery and recovery. Here, we present a case of a 24-year-old male who developed post-intubation tracheal stenosis. Repeated bronchoscopic dilatations were done for the same, but the symptoms persisted. Hence, the patient was planned for tracheal resection and anastomosis, the definitive surgical management. In this case report, we have discussed the anesthetic management of the same.

## Introduction

Management strategies for tracheal stenosis have advanced beyond the initial methods, such as repeated dilatations or the use of local and systemic steroids. Diagnostic measures, such as bronchoscopy, along with palliative techniques like dilation/stenting and endoscopic laser ablation, are available. Complex surgical procedures like tracheal resection and anastomosis can be employed for a definitive solution. Given the complexities and associated risks due to the shared airway and altered airway anatomy, a multidisciplinary approach is essential [1]. Stenosis close to the glottis is managed with a Montgomery T-tube. The strategies planned are based on recent bronchoscopic findings [2]. Interventional bronchoscopic management plays an important role in the treatment of benign tracheal stenosis, whereas complex stenosis needs a multidisciplinary approach and often requires surgical intervention [3]. The difficult airway cart and all the extra airway equipment, such as the Montgomery T-tube and flexometallic tubes, that could be required should be checked and kept ready.

## Case presentation

**Figure 1:**
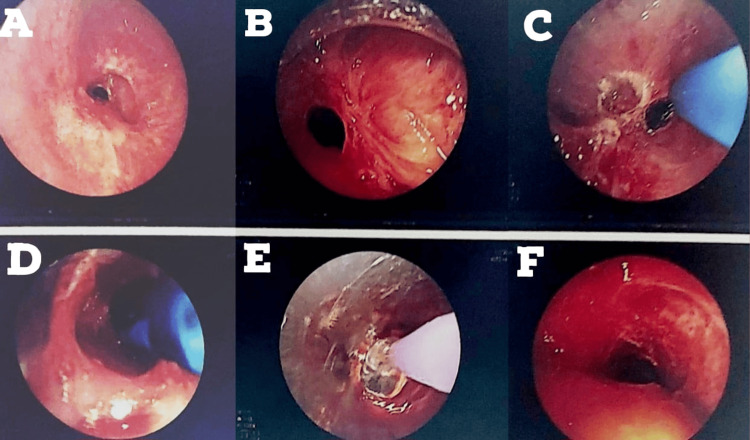
Bronchoscopy image dated July 28, 2022 (A) This figure shows stenosed trachea; (B)-(E) Sequential dilatations were done; (F) This figure shows dilated trachea

A 24-year-old male presented with tracheal stenosis due to organophosphate poisoning, which is the subject of this case. The patient came with complaints of breathlessness on exertion (NYHA grade 2) for three to four months, associated with complaints of cough with expectoration. The symptoms were not associated with fever and cold. The patient gave a history of organophosphate poisoning in July 2022, after which he was intubated and kept in the ICU for eight days. Post extubation (day 6), the patient started complaining of difficulty in breathing on exertion (NYHA grade 2), persistent coughing, and difficulty in coughing up the mucus. The patient was given an intravenous injection of effcorlin and was nebulized with duolin (ipratropium bromide and levosalbutamol respirator solution). CT thorax was performed on July 12, 2022, which showed subglottic stenosis with significant luminal narrowing as mentioned on the discharge card from that hospital (films were not available with the patient). On July 28, 2022, the patient underwent a rigid bronchoscopy procedure under general anesthesia with balloon dilatation for the same. Sequential dilatation was done, as shown in Figure 1.

**Figure 2:**
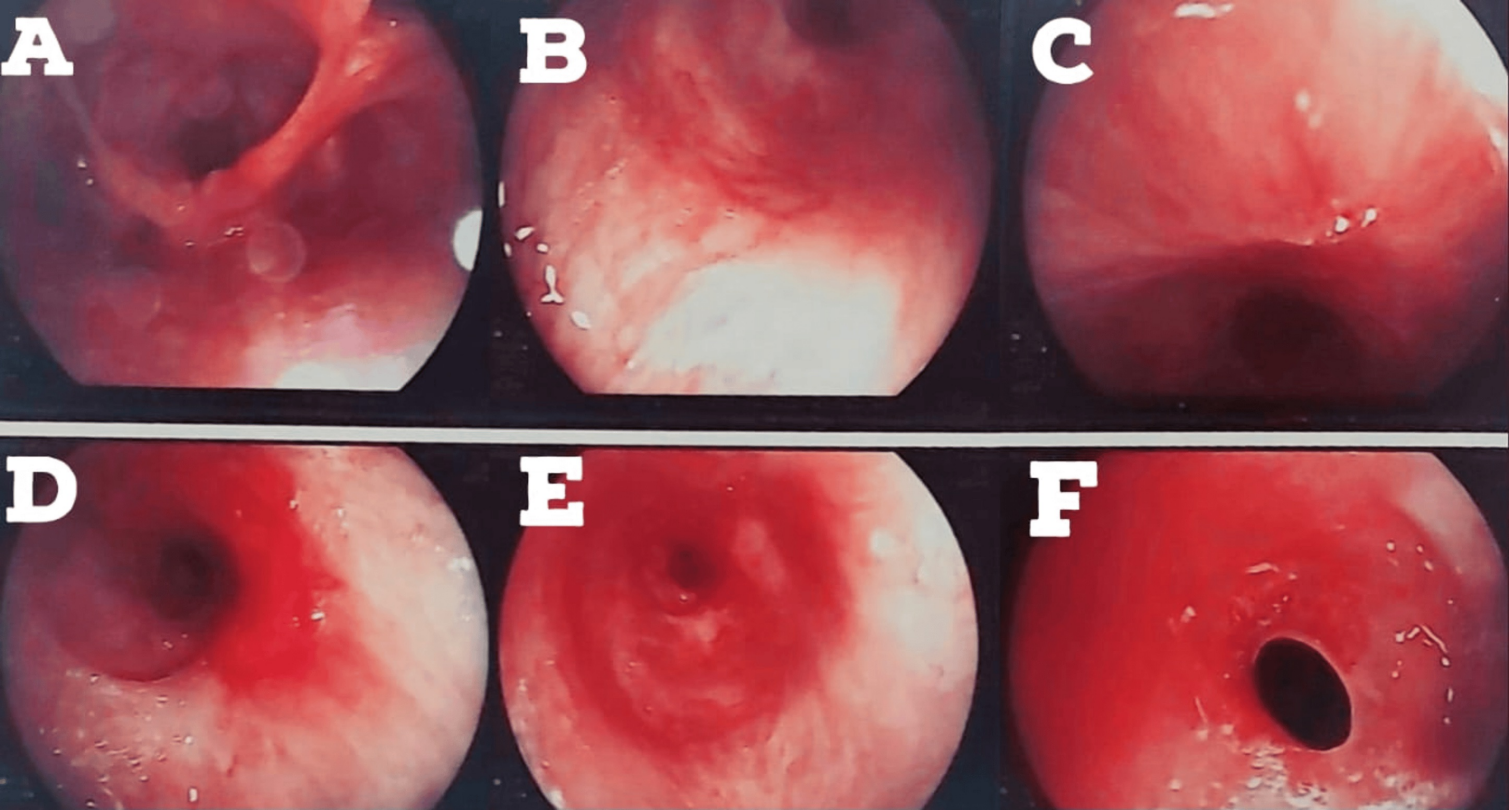
Bronchoscopy image dated August 24, 2022 (A)-(F) Trachea was found to be restenosed below the vocal cords, and silicon tracheal stenting was advised

The patient again started to develop breathlessness and excessive coughing since August 17, 2022. A bronchoscopy was done on August 24, 2022. The patient developed restenosis below the tracheostomized site, as shown in Figure 2. Silicon tracheal stent was advised (due to continuous requirement of bronchoscopic dilatations), and it was done in December 2022. 

The patient was admitted to our institute on January 1, 2023, and was planned for tracheal resection and anastomosis. On examination, the patient was vitally stable, with a heart rate of 88 bpm (beats per minute), blood pressure of 120/80 mmHg, and maintaining saturation of 100% (via rigid tracheostomy tube (TT)) on room air. Systemic examination revealed equal and clear bilateral chest air entry, and normal heart sounds were noted. The patient demonstrated good effort tolerance. The patient had a three-finger mouth opening and Mallampati grade 1. The analyses at the time of admission are mentioned in Table 1.

**Table 1:** Relevant routine investigations WNL: Within normal limits

Parameters	Patient values	Reference range
Hemoglobin	14.30 g/dL	13.2-16.6 g/dL
Total leukocyte count	8500/µL	4000-10000/µL
Platelets	310000/µL	150000-410000/µL
Urea	27 mg/dL	17-49 mg/dL
Creatinine	1.03 mg/dL	0.6-1.35 mg/dL
Prothrombin time	11.6 seconds	11-13.5 seconds
International normalized ratio	1.02	0.85-1.15
Liver function test	WNL	-
Serology	Non-reactive	-
Random blood sugar	94 mg/dL	70-140 mg/dL
Serum sodium	139 mmol/L	136-145 mmol/L
Serum potassium	4.2 mmol/L	3.50-5.10 mmol/L

**Figure 3:**
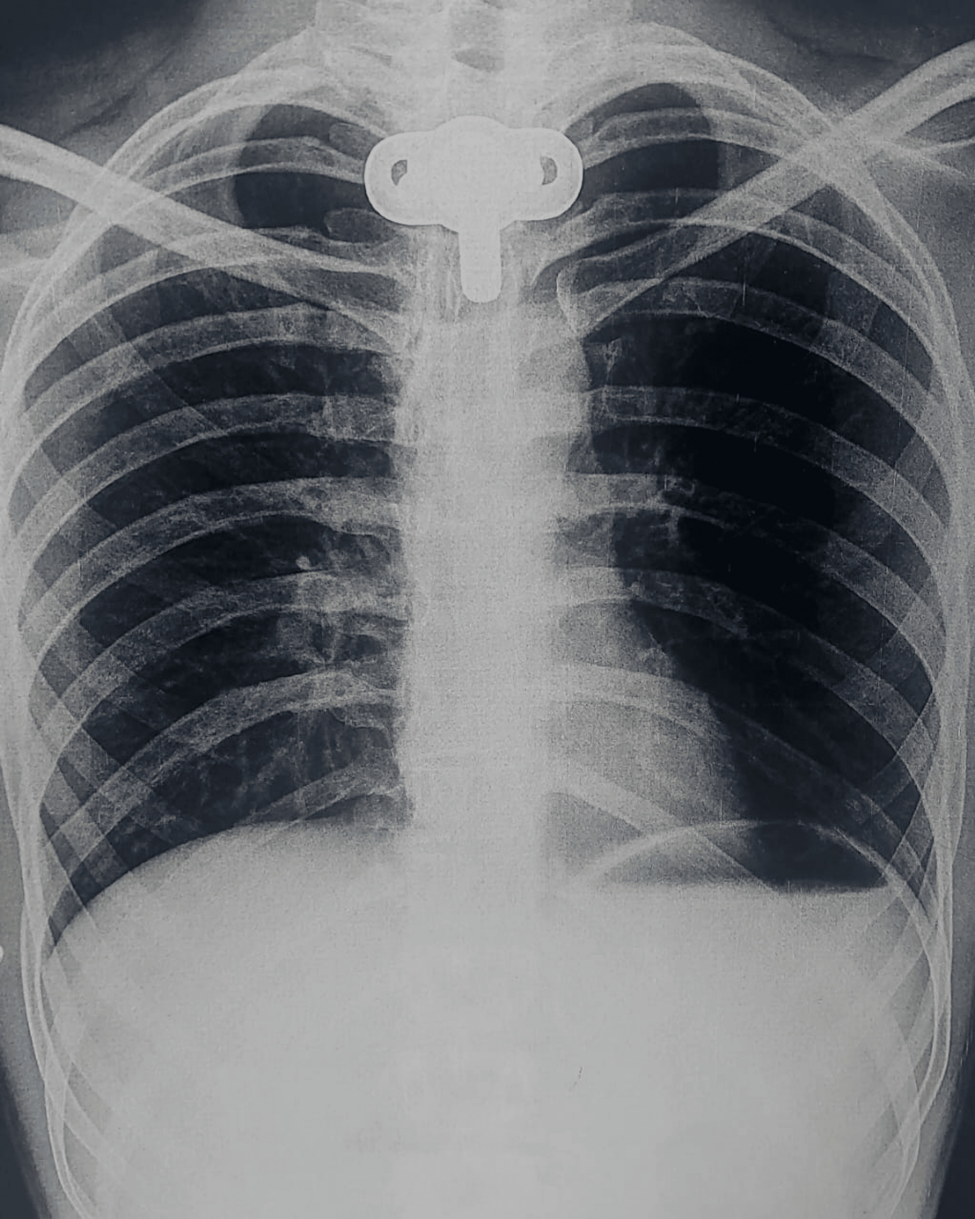
Chest X-ray on the day of admission Chest X-ray showed hyperinflated lung fields

**Figure 4:**
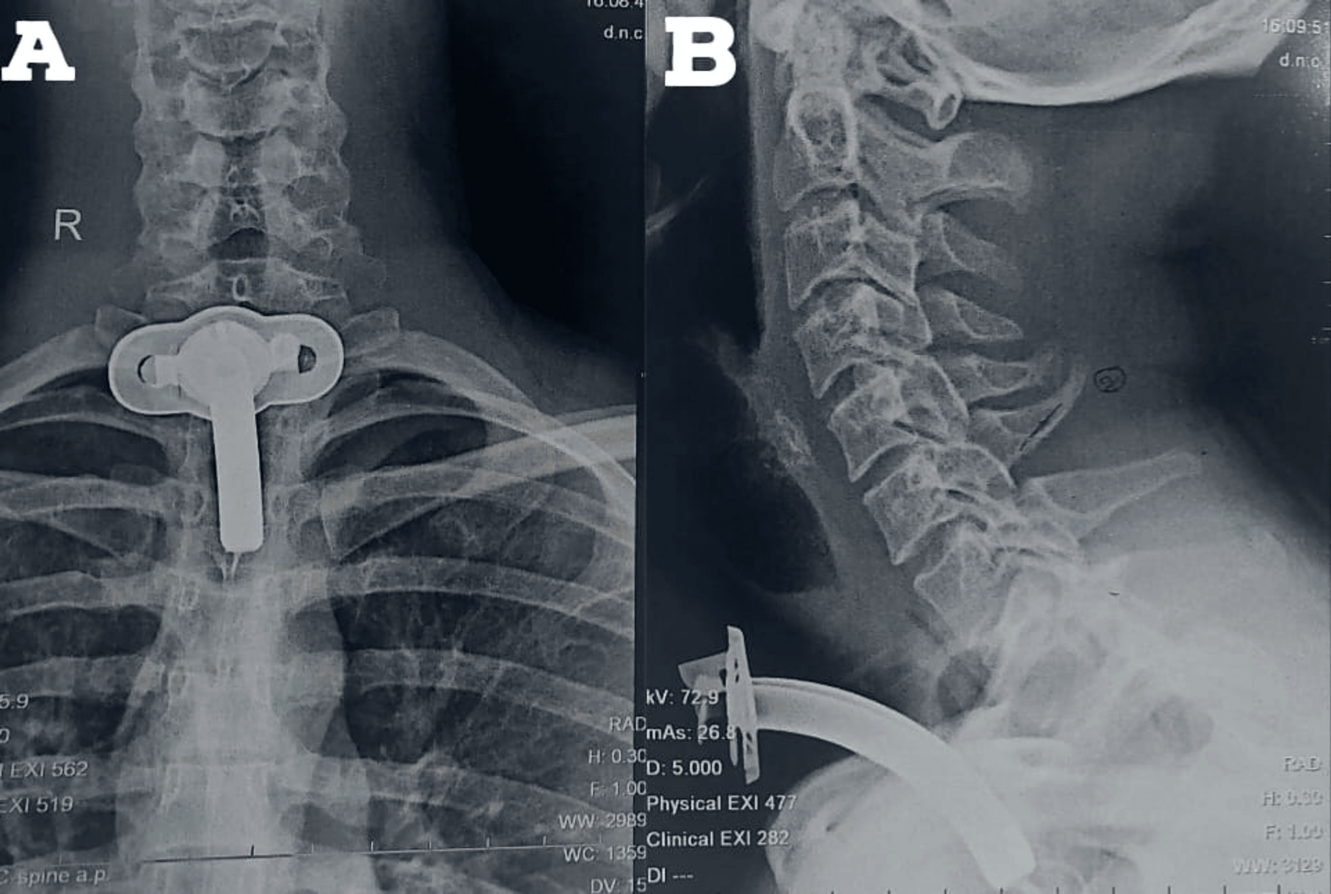
(A) X-ray cervical spine AP; (B) X-ray cervical spine lateral X-ray of the cervical spine showing tracheostomy site below C7 level, where the trachea is stenosed

**Figure 5:**
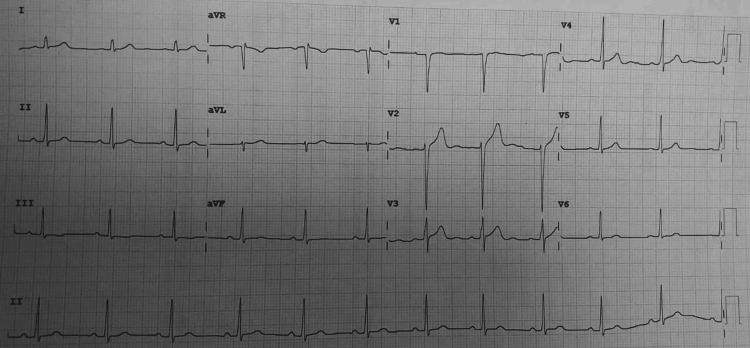
ECG on the day of admission depicting normal sinus rhythm

All laboratory investigations were within normal limits. Chest X-ray done was suggestive of hyperinflated lung fields, as shown in Figure 3. Also, Figure 4 depicts an X-ray neck film that showed a tracheostomy site just below the C7 level, where stenoses were present. ECG displayed normal sinus rhythm, which is presented in Figure 5. A 2D echo indicated an ejection fraction (EF) of 60%, with all other findings within normal limits.

Intraoperative management

The patient was assessed in the preoperative room. Two wide-bore intracaths were secured in both hands of the patient. The patient was administered an antibiotic and an injection of pantaprazole intravenously half an hour prior to incision time. The patient was taken inside the operating theatre. All the non-invasive monitors were attached. The patient came to the operation theatre tracheostomized with rigid metal TT. It was changed to a 7 mm cuffed PVC TT intraoperatively. The patient was then preoxygenated with 100% oxygen, and premedications were given: an injection of glycopyrrolate 0.2 mg IV followed by an injection of fentanyl 100 mcg. The patient was induced with an injection of propofol 100 mg. Muscle relaxant injection vecuronium was administered (loading dose of 6 mg), and after three minutes of manual ventilation, the patient was placed on volume control mode. An inhalational agent, sevoflurane, was started along with a combination of air and oxygen. Now, the patient was handed over to the surgeon. The incision was made below the tracheostomy site. In mid-surgery, since the flange of the 7 mm TT was becoming a hindrance, the 7 mm cuffed TT tube was replaced with a 7.5 mm flexometallic tube. The cuff was inflated, and the leftover length of the flexometallic tube was stitched to the patient’s skin to secure the airway properly, allowing the surgeons to prolong the incision for better manipulation.

**Figure 6:**
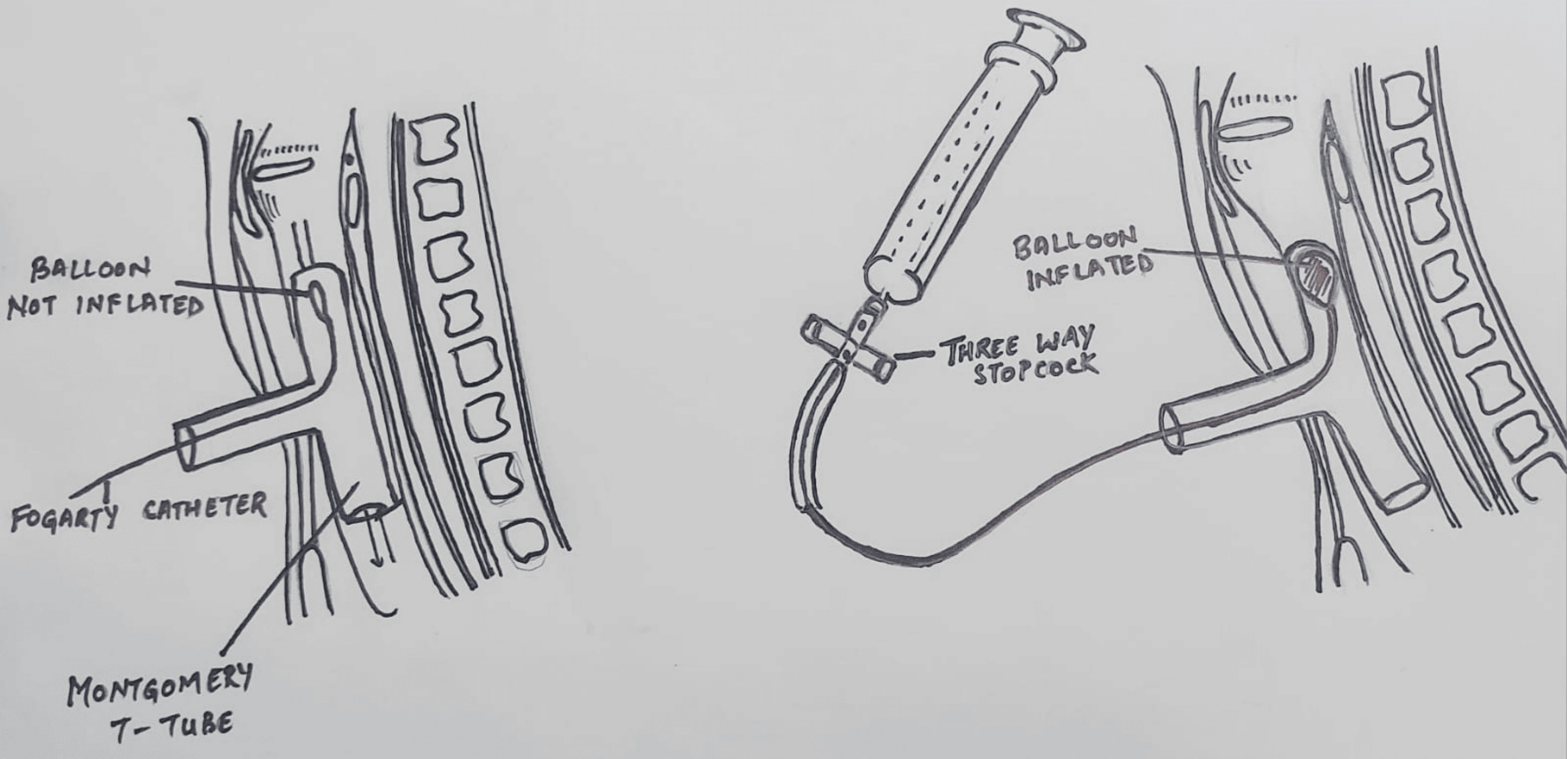
Montgomery T-tube and Fogarty catheter arrangement The figure is a self-illustration showing the placement of the Montgomery T-tube and Fogarty catheter used during tracheal reconstruction surgery inside the trachea. Image credit: Dr. Ipshita Garg

**Figure 7:**
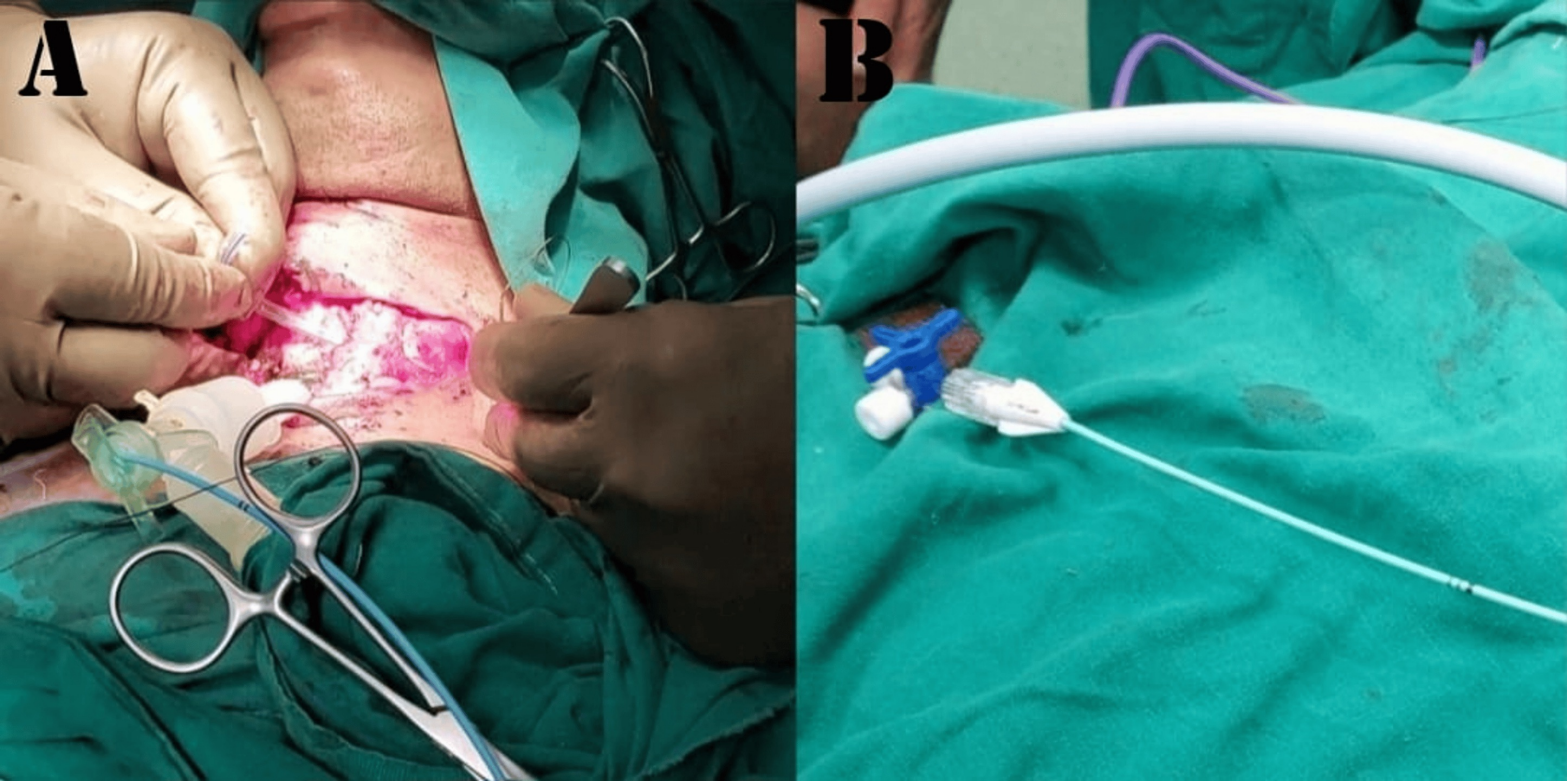
Image showing the inflated end of the Fogarty catheter coming out through the suction port of the catheter mount (A) Montgomery T-tube connected to endotracheal tube connector which is further connected to T-piece; (B) The other end of Fogarty catheter blocked with a three-way blocker

Tracheal resection and anastomosis were performed. During the procedure, the patient received four cycles of apnea, with a calculated time span as per the surgeon's requirement for better manipulation and successful tracheal anastomosis. A 12 mm Montgomery T-tube was taken, and a 6 mm Fogarty catheter was passed in one of the tracheal ends, and its cuff was inflated. After removal of the flexometallic tube, this arrangement was placed in the non-anastomosed segment of the trachea, with the Fogarty (cuff inflated) end placed towards the oropharyngeal side to avoid any air leak from that side. This made us achieve a secured tracheostomized airway using a Montgomery T-tube, wherein the outsourced oxygen will directly be supplied to the respiratory passage without any leaks from the oropharyngeal side. At the same time, the Montgomery T-tube would prevent the reconstructed trachea from collapsing and fibrosis, while the trachea takes its time to heal after surgery. The Montgomery T-tube was connected to a 7 mm ET tube connector, which was further connected to a catheter mount connected to ventilator circuits while the closure was still pending. The Fogarty catheter was passed inside this entire arrangement, and its other end was taken out from the suction port of the catheter mount, and the suction port was closed against this resistance. The patient was able to maintain good tidal volume with minimal leaks, and the free end of the Fogarty catheter was connected to a three-way to prevent air leaks from the cuff, as depicted in Figure 6. Figure 6 is a self-illustration that shows the Montgomery T-tube and Fogarty catheter placement inside the trachea that was used during the surgery. Figures 7A-7B are the intraoperative images of the above-mentioned placement.

The patient was given an injection of paracetamol 1 g, an injection of tramadol 50 mg, and an injection of diclofenac 75 mg intravenously during the surgery for adequate pain management. Surgeons, while closing, used guardian sutures to maintain the flexion of the neck and to stabilize the anastomosis. Twenty minutes before the end of the procedure, the patient received 4 mg of ondansetron intravenously. In the end, the patient was reversed after confirming adequate tone and breathing efforts. The catheter mount was connected to a T-piece, and the patient was shifted to ICU for further observation.

Two days later, the Fogarty catheter was removed, and the patient could breathe both from the T-tube and through the nose. Again, two days later, the patient was taken on a trial for breathing nasally only by closing the Montgomery T-tube extratracheal end and successfully maintaining 100% saturation on room air. So, the extratracheal end was closed, and the guardian suture was removed. The patient was able to vocalize and breathe nasally. He was discharged after a few days of observation with the T-tube in situ. 

## Discussion

Adult tracheal stenosis results mainly from inflammatory lesions (post-intubation, traumatic, and infectious) or tumors. Even though the incidence of post-intubation stenosis has decreased following the introduction of high-volume, low-pressure tracheal tube cuffs, it very much remains the commonest cause. Symptoms are nonspecific; cough and dyspnea on exertion are the first to develop. Dyspnea on exertion is the most common presentation due to a 50% reduction in tracheal diameter [4]. Slowly, it may as well proceed to dyspnea at rest. Our patient presented to us with complaints of dyspnea on exertion associated with cough and expectoration post-tracheal stenosis.

**Figure 8:**
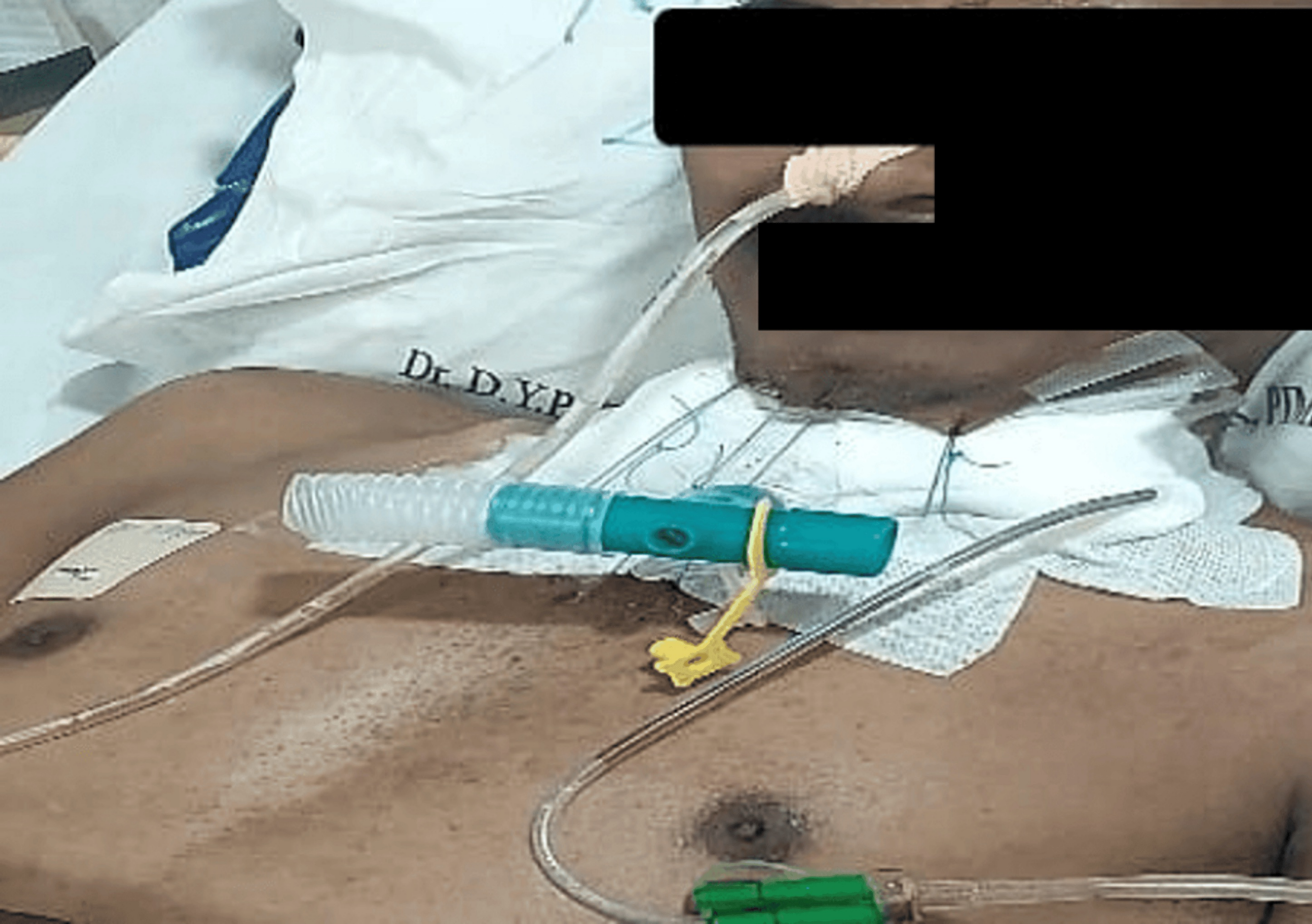
The guardian suture

The treatment of such conditions, where the very airway is compromised, aims at cure or at least palliation. Tracheal dilation with or without stenting, laser ablation, tracheostomy, or resection-anastomosis surgery are the treatment options available. The location and extent of stenosis should be confirmed, and accordingly, a plan of action should be made and discussed with the surgeons. Pre-surgery assessment holds significant importance. Adequate time must be allowed between bronchoscopy and surgery for tissue edema to settle before posting the patient for definitive surgery [5]. It's crucial to ensure the patient's pulmonary and cardiac status are optimized and thoroughly evaluated before scheduling the surgery. Also, the patient should be counseled regarding the guardian suture that limits neck extension on waking up after surgery, as shown in Figure 8. We made sure that our patient was well updated on the plan of anesthesia, the plan of surgery, and the post-operative anticipated guardian suture to limit neck extension, and well-informed consent was obtained regarding the same.

A number of ventilation strategies exist for patients with T-tubes during tracheal resection. The technique of upper limb occlusion by Fogarty catheter may be more secure compared to a throat pack or supraglottic airway. The main advantage of the T-tube includes the preservation of normal respiration and phonation with minimal tissue reaction to the silicon material post-surgery [6]. We followed this novel technique of antegrade passage of a Fogarty catheter through the mouth, guided by a suture, to the upper limb of the T-tube in our patient [7], which ensured that the leak was as minimal as possible and that the patient received the required tidal volume intra-operatively. 

Complications following tracheal resection and anastomosis or laryngotracheal resection and reconstruction can be broadly categorized as either anastomotic or non-anastomotic. Anastomotic complications encompass the formation of granulation tissue, tracheal restenosis, various levels of anastomotic separation, and the development of fistulas to nearby structures such as the innominate artery (tracheoinnominate fistula) and esophagus (tracheoesophageal fistula). Non-anastomotic complications specific to upper airway reconstruction include laryngeal edema and glottic dysfunction, affecting either phonation or swallowing. Complications following tracheal surgery are rare but must be addressed promptly and effectively when they arise to prevent major morbidity [8]. 

## Conclusions

Anesthesia for tracheal resection is a challenging task. Anesthetists strategize the anesthetic management according to the patient’s airway pathology, comorbidities, and surgical preference. Multidisciplinary teamwork and good communication with the surgeons are the keys to its success. Hence, with proper planning and good communication with our surgeons, we successfully executed this procedure. During the entire intraoperative procedure and postoperatively, the patient stayed vitally stable. Follow-up surgery happened seven months later to remove the Montgomery T-tube. As of today, the patient has a repaired trachea with no need for bronchoscopic dilatation and normal vocalization and breathing. Therefore, it can be concluded that this procedure has a high success rate and provides a better quality of life.
